# The House of Steinway

**Published:** 1875-06

**Authors:** 


### THE HOUSE OF STEINWAY.

In bringing our series of articles on the House of Steinway to a close, we deem it proper to make some allusions to the factory in which the most perfect pianos in the world are constructed. The great building occupies the entire front of the block extending from Fifty-Second to Fifty-Third Streets, on Fourth Avenue, New York City, and extends down both streets to a distance of 165 feet. A wing extends 100 feet still further down Fifty-Third Street, giving the great factory a street and avenue frontage of 631 feet.

The building is of brick, five stories high, of modern Italian style, and built in the most substantial manner. On the same block are two independent buildings, two stories in height, the dimensions of which are respectively 40 by 78 feet, and 100 by 20 feet,—the lower floors of which are devoted to the steam drying-rooms and the packing-box factory. In the upper rooms of these buildings all the actions and dampers are manufactured by the most skillful workmen to be obtained, aided by a series of the most perfect and ingenious machinery that exists for the construction of these parts.

The floors of the factory buildings have a surface of 160,480 square feet. In the rear of the buildings there is an open space of ground containing an area of 40,000 square feet, on which 3,000,000 feet of lumber are constantly stored in the open air, for seasoning purposes,—each separate piece of which is exposed to all the atmospheric changes for two years, and then kept in the steam drying-rooms for three months prior to being used. These drying-rooms are divided into five compartments, each of which contains about 80,000 feet of timber, so that about 400,000 feet are constantly under the process of kiln-drying. Each of the compartments is heated by 2,000 feet of steam-pipe. Beneath the yard alluded to, there are fireproof vaults for the storage of coal, and here also are placed four steam-boilers, of the aggregate power of 320 horses, by which the necessary amount of steam is generated for the 70,000 feet of pipe used in heating the drying-rooms, as well as supplying heat for the workshops and driving three steam-engines of respectively 125, 50, and 25 horse-power,—these, in turn, putting in motion no less than 102 different machines. These machines are said to equal, in power of production, not less than 500 skillful workmen.

This vast manufacturing business is divided into eighteen departments, each of which is placed under the control and constant personal inspection of a skilled foreman,—these, in turn, being controlled by a head foreman. No workman is permitted to work at more than one branch of the business; thus, from the fact that every workman is continually making only one and the same article, he achieves an absolute perfection in his work, unattainable in small factories, where such strict subdivision of labor can not exist. Again, in this great and strictly adhered to division of labor, the article, until it is finally completed, passes through the hands of a number of different workmen, none of whom receive it from the previous workman in that stage of manufacture unless it is perfectly faultless in every respect.

The control of the factory, the warerooms, the various purchases, is under the direct personal supervision of the members of the firm of Messrs. Steinway & Sons. All inventions and changes in the manufacture of pianos, and all other important business acts, are the result of common consideration and debate among the members of the firm, and to this harmonious co-operation and unanimity of action, a large proportion of the unexampled success which the firm has achieved may be attributed.

Among all the great industries of New York, it is doubtful if there is one which more fully than this exhibits the power of long-continued and intelligent effort. From the smallest beginings this famous firm has gone onward to a prosperity never before attained in the same line of business. Its products have been welcomed in all lands under the sun. Its fame is as wide as the science of music, to which its marvellous contributions have given a quickened impulse and a more exalted life. It is only just to say, what all New York will cheerfully admit, that among the leading establishments of the metropolis, none occupies a more exalted plane, or is more firmly based in the esteem alike of artists and the business world than The House of Steinway.

---

Relaxations, recreations, amusements, and pastimes, which tend to stimulate the passions unduly, excite impure emotions, and corrupt the heart, are to be strictly avoided.

---

All flowers will droop in absence of the sun that waked their sweets.

